# Interconnected pathways of social competence and resilience among foster care adolescents: Self-esteem acts as a potential mediator

**DOI:** 10.1016/j.mex.2025.103411

**Published:** 2025-05-31

**Authors:** Sandhiya Priyadarshini D, Tony P. Jose

**Affiliations:** Department of Social Sciences, School of Social Sciences and Languages, Vellore Institute of Technology, Vellore, India

**Keywords:** Mediation model, Direct effect, Indirect effect, Hayes' process macro, Foster care adolescents, Pearson’s correlation, Mediation analysis, Path analysis of direct and indirect effect

## Abstract

Foster care adolescents frequently experience heightened emotional and behavioral challenges. Although prior research has explored the link between social competence and resilience, the interplay of these factors specifically among foster care adolescents warrants further investigation. This study examined the relationships between self-esteem, social competence, and resilience in a sample of 200 adolescents residing in foster care homes. We hypothesized that self-esteem would mediate the connection between social competence and resilience. Participants were assessed using standardized measures of self-esteem, social competence, and resilience. The results indicated significant positive correlations among all three variables. Statistical analyses confirmed that self-esteem partially mediated the association between social competence and resilience. Social competence had both direct and indirect effects on resilience through its influence on self-esteem. The model accounted for 5.8 % of the variance in self-esteem and 23.7 % of the variance in resilience, emphasizing the interconnected nature of these constructs. These findings imply that interventions targeting these constructs may enhance well-being in foster care adolescents.

• Data were collected using standardized psychological scales.
• Correlational and mediation analyses examined relationships, focusing on self-esteem's mediating role.
• This study emphasizes the need for interventions that target these constructs to improve well-being in foster care adolescents.

## Graphical abstract

**Figure:**
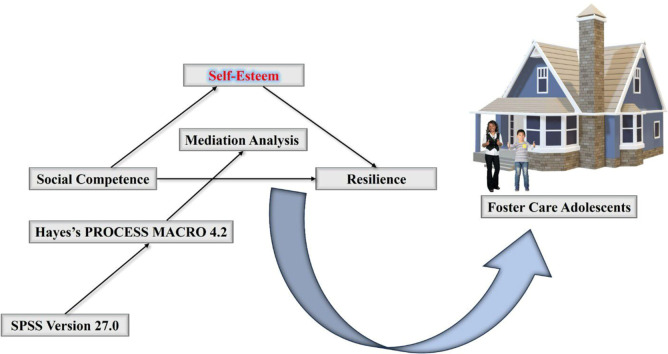

Specifications table

**Table:** 

Subject area:	Psychology
More specific subject area:	Positive Psychology
Name of your method:	Pearson’s correlation, Mediation analysis, Path analysis of direct and indirect effect
Name and reference of original method:	Hayes' MACRO tool in PROCESS 4.2 of SPSS version 27.0
Resource availability:	https://www.ibm.com/support/pages/downloading-ibm-spss-statistics-27 https://processmacro.org/download.html

## Background

Foster care homes are a critical part of the child welfare system, providing temporary residential placements for young people who cannot remain with their families. These environments aim to nurture children and adolescents until they can either return to their families, be adopted, or begin living independently [1]. Individuals enter the foster care system due to various traumatic experiences, which makes them particularly vulnerable to psychological distress. Adolescents in these settings often face significant emotional, behavioral, and psychosocial challenges stemming from adverse family circumstances such as poverty, abuse, or parental loss, resulting in higher risks of mental disorders compared to their peers [[2], [3], [4]].

The challenges experienced by foster care adolescents extend beyond immediate emotional difficulties. Past traumas like neglect and unmet needs significantly impact their development, leading to struggles in education, employment, health, and social adjustment that frequently persist into adulthood [[5], [6], [7]]. Research consistently demonstrates that foster children exhibit more emotional and behavioral problems than those raised in biological families, with gender-specific patterns showing boys more prone to behavioral issues while girls tend to experience more emotional difficulties [8]. These problems, including anxiety, depression, low self-esteem, diminished happiness, and aggression, highlight the critical need for enhanced support systems within foster care environments [[9], [10], [11], [12], [13]].

Social competence is widely recognized as a vital protective factor in promoting the mental health and well-being of adolescents, particularly those in vulnerable situations such as foster care. It encompasses a variety of interpersonal skills, including effective communication, empathy, conflict resolution, and self-expression, which enable individuals to establish and sustain positive relationships. Social competence—the ability to effectively navigate social relationships—encompasses essential skills such as communication efficacy, self-advocacy, empathetic understanding, and prosocial behavior patterns [14]. Adolescents with strong social skills are better able to manage interpersonal difficulties, alleviate psychological distress, and achieve greater emotional stability.

When adolescents develop strong social competencies, they experience multiple positive developmental outcomes, including enhanced emotional regulation, stronger academic achievement, more effective coping mechanisms, and increased positive self-regard [15,16]. These competencies also significantly influence self-perceptions by fostering a sense of capability and social connection, which can boost self-esteem. Moreover, social competence enhances resilience by enabling adolescents to navigate stressors more effectively, seek appropriate support, and maintain adaptive coping strategies. For foster care adolescents specifically, these competencies may serve as a critical foundation for building resilience. Thus, social competence works both directly and indirectly to strengthen well-being, making it a crucial element in understanding how self-esteem mediates the relationship between social functioning and resilience in foster care adolescents [17].

Resilience is crucial for maintaining well-being, especially when individuals encounter adversity. Although definitions of resilience may differ, it is commonly understood as a dynamic personal characteristic that allows individuals to adapt positively to stress and maintain emotional equilibrium [[17], [18], [19]]. Resilience, defined as the ability to thrive despite challenges, involves not only bouncing back from difficulties but also maintaining psychological equilibrium under adverse conditions [20,21]. Among adolescents, internal protective factors such as self-esteem have been identified as key contributors to resilience.

Resilient adolescents typically possess strong social skills, a sense of independence, effective problem-solving strategies, and a hopeful outlook [22]. Research indicates a reciprocal relationship between these two constructs [23,24]. High self-esteem is known to improve an adolescent’s ability to cope with challenges, and resilience itself can reinforce and elevate self-esteem. Moreover, resilience development is strengthened by supportive assets like meaningful relationships, problem-solving abilities, and healthy self-esteem. A particularly important protective factor for adolescents is a deep connection with a nurturing adult who provides unconditional acceptance and support, thereby fostering both social competence and resilience [25]. For example, resilient adolescents are more likely to experience greater life satisfaction and reduced psychological distress, with self-esteem serving as a key mediating mechanism. This interaction highlights the potential for a two-way relationship, where resilience strengthens self-esteem, and self-esteem supports the development of greater resilience [[26], [27], [28]]. Understanding this dynamic is particularly relevant when examining how self-esteem mediates the connection between social competence and resilience in foster care adolescents.

Self-esteem emerges as a particularly influential factor in foster adolescents' well-being, which is defined as a subjective evaluation of one's own worth that begins developing during childhood, equips individuals with the resilience necessary for navigating life's challenges [29,30]. However, adolescence—already characterized by heightened self-evaluation [[31], [32], [33]]—becomes especially difficult for foster care adolescents whose sense of worth is frequently undermined by traumatic experiences of family separation, placement instability, and the stigma associated with foster care [[34], [35], [36], [37], [38]]. Consequently, these adverse experiences increase their vulnerability to diminished self-worth and various mental health issues, including depression, substance abuse, and delinquent behavior [[39], [40], [41]].

The interplay between self-esteem, social competence, and resilience forms a complex relationship that warrants careful examination, particularly among foster care adolescents. Self-esteem plays a pivotal role in the intricate interplay of social competence and resilience, particularly among adolescents navigating the complexities of foster care. When foster care adolescents possess healthy self-esteem, they develop positive self-regard that empowers them to engage confidently in social situations, express their needs effectively, and navigate interpersonal challenges constructively. Conversely, diminished self-esteem can lead to feelings of inadequacy, self-doubt, and social anxiety, potentially causing withdrawal from social interactions. Self-esteem serves as a cornerstone of resilience, enabling adolescents to bounce back from setbacks and maintain optimism despite adversity. Those with high self-esteem tend to view challenges as growth opportunities and persevere through difficulties, while low self-esteem can lead to feelings of helplessness and diminished coping capacity. The interconnection between social competence and self-esteem is particularly significant, as adolescents who perceive themselves as socially adept tend to develop stronger self-worth, enhancing their ability to cope with stress. This highlights the importance of interventions that target both social skills development and self-esteem enhancement for foster care adolescents, prioritizing opportunities for them to develop mastery, build positive relationships, and receive validation for their strengths.

While self-esteem plays a significant role in adolescent development, particularly within foster care, it may not be the primary driving factor in the relationship between social competence and resilience. Instead, self-esteem may function more effectively as a mediating factor. An adolescent's ability to successfully navigate social situations can influence the development of their self-esteem. Specifically, positive feedback garnered from social interactions—acceptance, peer engagement, and a sense of social efficacy—contributes to stronger self-worth. This perspective aligns with developmental theories, such as the *sociometer theory*, which posits that self-esteem acts as an internal gauge of social acceptance and belonging [42]. According to this theory, self-esteem arises from our perception of how others view us; feeling valued and accepted by peers' bolsters self-esteem, while perceived rejection diminishes it. Consequently, healthy self-esteem promotes adaptive coping mechanisms, improved emotional regulation, and greater resilience. Therefore, while the importance of self-esteem remains, its impact on resilience is likely dependent on foundational social competencies. Ultimately, resilience requires practical skills and stable, supportive relationships in addition to self-esteem.

Understanding these interconnected pathways is particularly crucial in the foster care context, where support systems may be inconsistent or limited. By exploring the relationships among social competence and resilience with specific focus on the mediating role of self-esteem—this research aims to contribute to a deeper understanding of the internal mechanisms that promote well-being among foster care adolescents. Quantifying these relationships within this vulnerable population, as this study intends, addresses a significant research gap and directly examines these dynamics within the foster care system—a population experiencing unique challenges. This underscores self-esteem's significant influence and delivers valuable insights to mental health professionals, caregivers, social workers, and policymakers, ultimately informing evidence-based educational programs and improving outcomes for at-risk youth. These insights can ultimately inform the development of targeted interventions that enhance both immediate adjustment and long-term outcomes for this vulnerable population [43].

## Method details

### Objectives

The primary main goal of this study is to investigate the connections between self-esteem, social competence, and resilience among foster care adolescents. More Specifically, it aims to examine whether self-esteem serves as a mediating factor between social competence and resilience in this population. This involves exploring how self-esteem influences both resilience and social competence.

This study posited two primary hypotheses:

• H_1_: Self-esteem is positively associated with social competence and resilience among foster care adolescents.
• H_2_: Self-esteem would be a significant mediator between social competence and resilience among foster care adolescents.

### Participants and procedure

Two hundred adolescents, aged 13–17, from foster care settings in Chennai, Tamil Nadu, were recruited from four foster care homes. Potential participants were approached directly through the head of foster care homes, and participation of foster care adolescents was voluntary.

Before data collection, the purpose of the study was explained to all institutional heads, and participants who obtained the necessary permissions. Informed consent forms were provided, reviewed, and signed by all participants. Ethical approval for the study was granted by the *“Institutional Ethical Committee for Studies on Human Subjects (IECH) of Vellore Institute of Technology, Vellore”*. The research study adhered to local laws and institutional guidelines. Participant confidentiality was guaranteed, and they were advised of their freedom to discontinue their involvement whenever they wish.

A convenience sampling method was employed, selecting participants readily accessible to the researcher. Data collection involved administering standardized psychological questionnaires. Participants were provided with the questionnaires and instructed to complete them independently. The estimated time for questionnaire completion was approximately 20 min. A total of 26 valid items of all questionnaires were collected.

### Instruments

The following standardized instruments were utilized.

The *“Rosenberg Self-Esteem Scale”*, a well-established assessment of self-esteem developed by Dr. Morris Rosenberg [44], employs a ten-item Likert scale that assesses positive and negative feelings about the self. Respondents rate their agreement with statements concerning self-worth and self-approval (for instance, “I feel that I’m a person of worth, at least on an equal plane with others”) assessed on a 4-point response scale from “Strongly Disagree” to “Strongly Agree”. Each item receives a score from 0 to 3, with “Strongly Disagree” being 0 and “Strongly Agree” being 3. The total score, representing the sum of individual item scores, falls between 0 and 30, with higher scores corresponding to elevated self-esteem. The RSES boasts high internal consistency, demonstrated by a Cronbach alpha 0.92. This scale has been widely used and validated across diverse populations and research settings.

The *“Perceived Social Competence Scale”*, a tool created by Anderson-Butcher et al. [45], was used in this study. It evaluates individuals perceived social competence. This six-item scale employs a 5-point Likert format, with responses ranging from “Not at All (1)” to “Very Much (5)”, for responses to statements like “I get along well with others.” Scores span from a minimum of 6 to a maximum of 30, where elevated scores indicate stronger social abilities and interpersonal skills. The original scale's initial Cronbach alpha was 0.87, indicating good reliability. The items' focus on prosocial behaviors and positive social interactions supports their face validity.

The *“Connor-Davidson Resilience Scale, a 10-item version”* developed by Campbell-Sills and Stein [46], was used to measure resilience. This shortened version maintains the original's psychometric strengths while offering a streamlined assessment. Employing a 5-point response scale (from “not true at all” to “true nearly all the time”), participants reflected on their experiences over the last month. Resilience scores, ranging from 0 to 40, directly correlated with higher ratings. The scale demonstrates reliability, as shown by its unidimensionality and high Cronbach's alpha 0.85. Furthermore, its construct validity reinforces its effectiveness as a measure of resilience.

### Data analysis

The Pearson product-moment correlation was computed using the statistical software IBM SPSS version 27.0 to explore the associations between the variables. Certainly, the researcher built the mediation model using Hayes' PROCESS 4.2 [47] to test the hypothesized model and mediation effects. Finally, indirect effects were examined using the Bootstrap method; we set 5000 bootstrap samples and 95 % confidence intervals.

## Method validation

### Analyzing the correlation between self-esteem, social competence, and resilience

Table 1 presents the correlational analysis between self-esteem, social competence, and resilience among adolescents in foster care homes. The results revealed statistically significant positive correlations between all three variables. Self-esteem was positively linked with both social competence (*r* = 0.241, *p* < 0.01) and resilience (*r* = 0.292, *p* < 0.01). This indicates that adolescents with higher self-esteem tended to exhibit greater social competence and resilience. Furthermore, a strong positive correlation (*r* = 0.448, *p* < 0.01) was observed between social competence and resilience, indicating that adolescents in foster care with higher levels of social competence tended to demonstrate greater resilience. These findings suggest that the interrelation of these three constructs positively influences the well-being of these foster care adolescents. Hence, the hypothesis (H_1_) stating that self-esteem is positively associated with social competence and resilience among foster care adolescents was accepted.

**Table 1:** Pearson Correlations among self-esteem, social competence, and resilience.

	M	SD	1	2	3
1. Self-esteem	18.97	3.430	1		
2. Social Competence	23.54	3.982	0.241**	1	
3. Resilience	26.18	6.476	0.292**	0.448**	1

Note:

⁎⁎ Correlation is significant at the 0.01 level (2-tailed).

### Mediation model: effect of self-esteem in the association between social competence and resilience

**Fig. 1:**
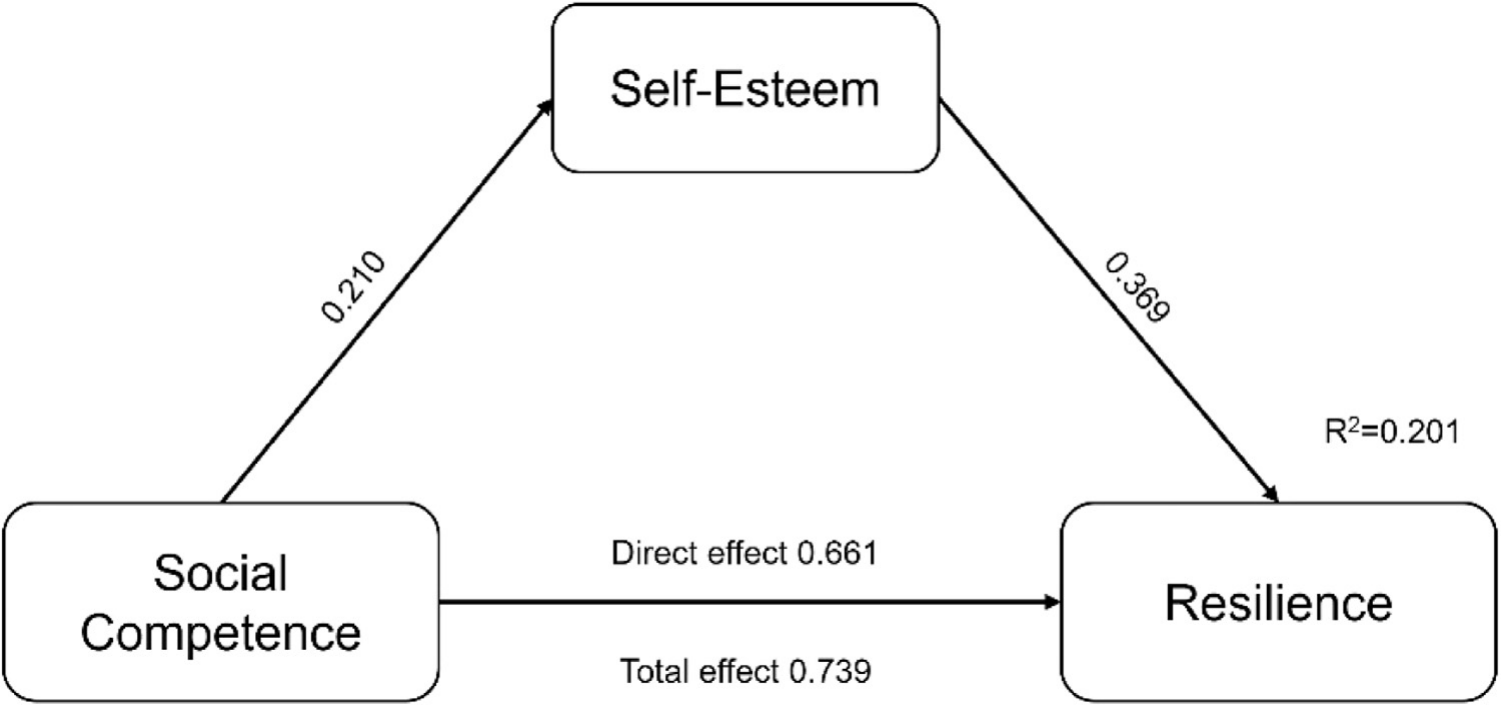
Model depicting self-esteem as a mediator in the association between social competence and resilience among foster care adolescents. Note: ***The correlation is significant at 0.001 (*p* < 0.001).

Fig. 1 illustrates the mediation analysis exploring self-esteem's influence on the association between social competence (independent variable) and resilience (dependent variable) in adolescents within foster care settings. Data analysis employed Hayes' PROCESS macro (version 4.2) within SPSS (version 27.0).

The analysis revealed a statistically significant positive link emerged between social competence and self-worth (effect size = 0.210, *p* = 0.001), indicating that higher social competence in foster care adolescents was linked to elevated self-esteem. Furthermore, social competence directly and positively influenced resilience (effect size = 0.661, *p* < 0.001), suggesting a greater likelihood of resilience among adolescents in foster care with stronger social skills. Similarly, self-esteem served as a Self-esteem was a positive indicator of resilience (effect size = 0.369, *p* = 0.003), associating higher self-esteem with increased resilience.

Social competence significantly predicted resilience (total effect size = 0.739, *p* < 0.001). Importantly, the analysis revealed that self-esteem partially mediated this relationship (indirect effect = 0.078, BootSE = 0.036, 95 % CI [0.018, 0.160]), implying that social competence fosters resilience partly by boosting self-esteem. Although social competence continued to directly influence resilience, the significant indirect effect confirms self-esteem's mediating role, as further supported by the completely standardized indirect effect (0.047, BootSE = 0.022, 95 % CI [0.011, 0.096]). Despite the statistically significant mediation, the substantial direct effect of social competence on resilience suggests other factors also play a role. The model captured 5.8 % of the variance in self-esteem and explained a considerably larger portion 23.7 % of the variance in resilience, demonstrating that social competence bolsters resilience both directly and indirectly via its positive effect on self-esteem in adolescents within foster care settings. Hence, the hypothesis (H_2_) stating that self-esteem is a significant mediator between social competence and resilience among foster care adolescents was accepted.

## Discussion

This study investigates the complex interplay of social competence, resilience, and self-esteem in foster care adolescents. The results consistently reveal a strong positive association among these factors, emphasizing the importance of social competence and self-esteem in fostering resilience within this vulnerable group. Furthermore, self-esteem plays a significant mediating role in the relationship between social competence and resilience. This finding underscores the importance of self-esteem as a crucial mechanism through which social competence influences resilience. While many studies have investigated self-esteem's mediating role in various academic contexts, this study uniquely focuses on its specific influence on the relationship between social competence and resilience specifically in foster care adolescents. This is a novel perspective, as some studies explore related constructs from different perspectives.

Primarily, this research study confirms a strong positive link between being socially competent (e.g., possessing effective communication skills and the ability to form positive relationships) and having high self-esteem. Similarly, prior studies carried out in Brazil and Northern Chile have established that individuals with greater perceived social competence demonstrate higher levels of self-concept and self-esteem. This association is especially crucial for adolescents in foster care because they often face challenging social environments, such as instability and limited social support. Because self-esteem is built through interactions, individuals who struggle with social competence may experience diminished self-regard [[48], [49], [50], [51]].

Adolescents who feel less socially competent often struggle with their self-image, leading to lower self-esteem and increased feelings of loneliness [52]. This is consistent with the positive relationship between perceived social competence and self-esteem found in this study. As adolescents' self-esteem increases, their perception of their social abilities likely improves as well. Social competence is vital for social and emotional development, particularly for foster care adolescents who often experience unstable relationships and lack of social support [53]. Research suggests that individuals with a positive view of their social skills tend to have higher self-esteem, as self-evaluation often involves assessing social and emotional functioning. These skills help adolescents form and maintain strong relationships, boosting their self-worth and promoting greater psychological resilience [54]. It is important to note that some research, though limited, has not found a significant connection between resilience and self-esteem in adolescents, a finding that differs from the conclusions of this study [55]. This inconsistency may stem from the unique circumstances of adolescents in foster care, whose experiences may shape the relationship between these factors differently than in other adolescent populations.

The mediational analysis uncovered a nuanced mechanism by which social competence influences resilience through self-esteem. While the direct effect of social competence on resilience is substantial, the indirect effect through self-esteem suggests that social skills contribute to psychological resilience by first enhancing an individual's self-perception. As the literature indicates, positive interpersonal experiences substantially shape self-perception, while perceived social competence significantly influences self-worth [56,57]. Therefore, fostering social competence can indirectly bolster resilience by strengthening self-esteem.

Understanding the mediating influence of self-esteem provides crucial insights into the relationship between social competence and resilience. Adolescents with higher social skills tend to develop stronger self-esteem, which in turn enhances their ability to overcome adversities. This finding is consistent with research in Turkey and England, which has demonstrated a significant association between social integration and competence, and resilience levels. Studies have shown that those who are less integrated, or excluded by their peers, have lower levels of resilience [15,58]. Conversely, those who perceive greater competence at the social level are more resilient because positive social bonds increase resilience [[48], [49], [50]]. This highlights the importance of creating supportive social environments for foster care adolescents.

Self-esteem is a critical protective factor for individual resilience [59,60], fostering confidence in navigating challenges, making autonomous decisions, and learning from mistakes. Consistent with prior studies, a strong positive link exists between self-esteem and resilience in adolescents within foster care settings [51]. This underscores the potential for interventions aimed at boosting self-esteem to significantly enhance resilience.

As a fundamental personality trait, self-esteem shapes the development of resilience, influencing how adolescents respond to adversity [18,61]. High self-esteem empowers them to handle stress and overcome challenges more effectively, promoting healthy coping strategies [62]. This is particularly salient for foster care adolescents, who often face emotionally difficult situations such as parental absence or unstable relationships. Adolescents with high self-esteem tend to be more emotionally stable, confident in their ability to manage difficulties, and likely to seek help from caregivers, teachers, or friends when needed [63,64]. Conversely, those with low self-esteem may resort to maladaptive coping mechanisms like avoidance, self-blame, or escapism [65]. Furthermore, higher self-esteem has been consistently associated with better mental well-being, improved emotional regulation, and a reduced risk of developing mental health problems [66]. These findings align with the results of numerous studies [[67], [68], [69], [70], [71], [72], [73]], demonstrating that self-esteem positively influences resilience. Adolescents with higher self-esteem tend to exhibit greater resilience and report better mental health and overall well-being. Furthermore, self-esteem serves as a mediating factor in the relationship between social competence and resilience among adolescents in foster care.

Well-being, in this context, is a vital aspect of overall health and quality of life, encompassing emotional, mental, and social dimensions. It goes beyond the mere absence of mental illness and represents a multifaceted concept that includes the positive aspects of mental health. Considering the unique and often profound challenges faced by adolescents in foster care, well-being is of utmost importance. *Ryff's theory of psychological well-being* offers a relevant framework for understanding the complex interplay of social competence, resilience, and self-esteem, particularly among adolescents in foster care. Researchers have conceptualized and highlighted several key components, including self-acceptance, autonomy, personal growth, environmental mastery, positive relationships with others, and a sense of purpose or meaning in life [74,75]. These components emphasize the significance of fostering a positive self-perception, cultivating meaningful relationships, and developing the capacity to navigate life's challenges. Three core dimensions directly align with this study’s constructs: self-acceptance, which reflects the foundation of self-esteem and the need for positive self-regard despite adversity; positive relations with others, corresponding to social competence and the ability to form meaningful connections; and environmental mastery, aligning with resilience through effective adaptation to challenging foster care environments. Ultimately, well-being promotes a sense of satisfaction with oneself and one’s connections with others [76].

The interconnected nature of these constructs suggests that intervention programs should focus on holistic approaches. By targeting social competence and self-esteem simultaneously, educators and caregivers can potentially enhance the resilience of adolescents in foster care. Franco et al. endorse this perspective, emphasizing the connection between strong social skills and beneficial outcomes like emotional regulation, academic success, and adaptive coping mechanisms [16]. Thus, a multifaceted approach addressing both social and emotional well-being is likely to be most effective.

To summarize, this study underscores the critical importance of social competence and self-esteem in developing resilience among adolescents in foster care homes. The findings suggest that improvement in one variable can positively influence the others, creating a potentially virtuous cycle of personal development and well-being. Recommendations for future research include longitudinal studies that can further explore the causal relationships between these variables and develop targeted intervention strategies to support adolescents in foster care environments.

## Limitations

This study has several limitations that warrant consideration. As a cross-sectional study collecting data at a single time point, it provides only a snapshot of participants' experiences and precludes causal inferences about the relationships between variables. The use of a convenience sample may limit the generalizability of the findings to the broader population of adolescents in foster care. Additionally, reliance on self-reported measures introduces potential biases, including common method variance and social desirability effects. The geographic focus on Chennai further restricts the applicability of the results to other regions and cultural contexts. Finally, the study focused on a limited set of variables; future research should consider incorporating a wider range of psychosocial factors that may influence outcomes in this population.

Future research should consider testing alternative models of mediation and causality. For instance, exploring whether self-esteem may serve as a foundational variable that influences resilience, which in turn impacts social competence, could provide deeper insights into developmental pathways. Longitudinal research designs would be especially valuable for clarifying the directionality of these relationships over time. Furthermore, future studies could examine the moderating role of contextual factors such as quality of foster care, peer support, and caregiver attachment in shaping these psychological outcomes. Cross-cultural studies could also illuminate how cultural norms and caregiving practices influence the development of self-esteem and resilience in foster care adolescents. By addressing these gaps, future research can build a more comprehensive understanding of how to best support the well-being of adolescents in foster care.

## Educational implications

The findings of this study offer valuable guidance for educational and caregiving professionals working with adolescents in foster care. Given the central role of self-esteem in mediating the relationship between social competence and resilience, it is essential to design interventions that target both social and emotional development. Educational institutions, foster care homes, and community programs should consider implementing evidence-based strategies to strengthen social skills and reinforce adolescents’ self-worth.

Research supports the effectiveness of structured social-emotional learning programs in enhancing peer relationships, emotional regulation, and self-efficacy [15,16]. Programs that include components like cooperative learning, peer mentoring, and adult guidance can significantly contribute to developing both social competence and self-esteem. For adolescents in foster care, adult mentors and educators can serve as consistent figures of support, helping to rebuild a sense of belonging and identity. Trauma-informed approaches and attachment-based interventions may also help foster care adolescents build secure relationships, which are critical for resilience [22,25].

By integrating these approaches into educational and foster care programs, stakeholders can create environments where adolescents feel valued, capable, and resilient. This not only promotes better psychological functioning but also enhances academic and social outcomes over time.

## Ethics statements

The studies involving human participants were approved by the Institutional Ethical Committee for Studies on Human Subjects (IECH) of Vellore Institute of Technology, Vellore. They were conducted in accordance with local legislation and institutional requirements.

## Informed consent

Informed consent was obtained from all participants.

## CRediT authorship contribution statement

**Sandhiya Priyadarshini D:** Conceptualization, Methodology, Software, Data curation, Writing – original draft. **Tony P. Jose:** Investigation, Software, Validation, Supervision, Writing – review & editing.

## Declaration of competing interest

The authors declare that they have no known competing financial interests or personal relationships that could have appeared to influence the work reported in this paper.

## Data Availability

The data that has been used is confidential.
